# A multi-institutional retrospective study of hyperthermic plus intravesical chemotherapy versus intravesical chemotherapy treatment alone in intermediate and high risk nonmuscle-invasive bladder cancer

**DOI:** 10.20892/j.issn.2095-3941.2020.0125

**Published:** 2021-02-15

**Authors:** Qiang Ruan, Degang Ding, Bin Wang, Chaohong He, Xuequn Ren, Zhenhua Feng, Zhigang Pang, Jin Wang, Xiangliang Zhang, Hongsheng Tang, Jiahong Wang, Qingjun He, Ziying Lei, Quanxing Liao, Jiali Luo, Shuzhong Cui

**Affiliations:** 1 Department of Abdominal Surgery, Affiliated Cancer Hospital & Institute of Guangzhou Medical University, Guangzhou 510095, China; 2 The First Affiliated Hospital of Jinan University, the First Clinical Medical College of Jinan University, Guangzhou 510630, China; 3 Department of Urinary Surgery, Henan Provincial People’s Hospital, Zhengzhou 450003, China; 4 Department of Urinary Surgery, Affiliated Cancer Hospital & Institute of Guangzhou Medical University, Guangzhou 510095, China; 5 Department of Urinary Surgery, Henan Cancer Hospital, Zhengzhou 450008, China; 6 Department of General Surgery, Huaihe Hospital of Henan University, Kaifeng 475000, China; 7 Department of Urinary Surgery, Gaozhou People’s Hospital, Maoming 525200, China; 8 Department of General Surgery, The Second Affiliated Hospital of Zhengzhou University, Zhengzhou 450014, China

**Keywords:** Nonmuscle-invasive bladder cancer, intravesical chemotherapy, hyperthermia, chemohyperthermia, retrospective study

## Abstract

**Objective:**

To compare the efficacy and safety of hyperthermic intravesical chemotherapy (HIVEC) and intravesical chemotherapy (IVEC) in patients with intermediate and high risk nonmuscle-invasive bladder cancer (NMIBC) after transurethral resection.

**Methods:**

We included 560 patients diagnosed with primary or recurrent NMIBC between April 2009 and December 2015 at 1 of 6 tertiary centers. We matched 364 intermediate or high risk cases and divided them into 2 groups: the HIVEC+IVEC group [chemohyperthermia (CHT) composed of 3 consecutive sessions followed by intravesical instillation without hyperthermia] and the IVEC group (intravesical instillation without hyperthermia). The data were recorded in the database. The primary endpoint was 2-year recurrence-free survival (RFS) in all NMIBC patients (*n* = 364), whereas the secondary endpoints were the assessment of radical cystectomy (RC) and 5-year overall survival (OS).

**Results:**

There was a significant difference in the 2-year RFS between the two groups in all patients (*n* = 364; HIVEC+IVEC: 82.42% *vs.* IVEC: 74.18%, *P* = 0.038). Compared with the IVEC group, the HIVEC+IVEC group had a lower incidence of RC (*P* = 0.0274). However, the 5-year OS was the same between the 2 groups (*P* = 0.1434). Adverse events (AEs) occurred in 32.7% of all patients, but none of the events was serious (grades 3–4). No difference in the incidence or severity of AEs between each treatment modality was observed.

**Conclusions:**

This retrospective study showed that HIVEC+IVEC had a higher 2-year RFS and a lower incidence of RC than IVEC therapy in intermediate and high risk NMIBC patients. Both treatments were well-tolerated in a similar manner.

## Introduction

Bladder cancer is the most common malignancy of the urinary system, and approximately 75% of bladder cancers are superficial and can be classified as carcinoma *in situ* [CIS, a highly aggressive type of nonmuscle-invasive bladder cancer (NMIBC)], Ta (confined to the urothelium), or T1 (invading the lamina propria)^1^. According to the degree of malignancy, NMIBC can be further classified as low grade (grades 1 and 2) and high grade (grade 3)^2^. More than 60% of all high risk patients experience recurrence within 1 year after diagnosis^3,4^. Transurethral resection (TUR) is the standard treatment for NMIBC, and despite the availability of complete TUR and the addition of adjuvant therapies, NMIBC has a strong tendency to recur; 52% of patients with high risk NMIBC will experience recurrence, and 20% (especially T1 patients) will progress to muscle-invasive bladder cancer within 5 years^5^.

Currently, intravesical chemotherapy (IVEC) is an acceptable treatment for intermediate- and high risk NMIBC after TUR^6,7^. Cytostatic drugs [e.g., mitomycin C (MMC), epirubicin, and thiotepa] and the immunomodulating agent, bacillus Calmette-Guérin (BCG), are commonly administered^8^. Intravesical instillation of BCG is effective in delaying or preventing recurrence, but it is associated with local and systemic side effects and has a percentage of recurrence of approximately 38% at 2 years post-TUR^9–11^. Regrettably, because of the worldwide shortage of BCG^12^, it is unavailable in many countries. Therefore, new effective approaches for the management of NMIBC need to be developed and clinically tested.

Hyperthermia has antitumor effects through a variety of mechanisms, including direct and immune-based cytotoxicity^13,14^, which can be further enhanced by the addition of chemotherapy^15,16^. In recent years, hyperthermic intravesical chemotherapy (HIVEC) has been used in clinical practice, with HIVEC with MMC as one of the most intensively studied. It has been reported that chemohyperthermia (CHT) controls disease progression and reduces local recurrence^17,18^. A retrospective study of 111 patients showed that patients treated with HIVEC had a recurrence percentage of 17% within 2 years, compared to 58% in those treated with chemotherapy alone (*P* = 0.0002)^19^.

In this study, we therefore reported the results of intermediate and high risk NMIBC patients who had been treated with IVEC or HIVEC+IVEC.

## Materials and methods

### Patients and the database

We collected data from 6 hospitals and established a database. These hospitals included Henan Provincial People’s Hospital, Henan Cancer Hospital, Huaihe Hospital of Henan University, Affiliated Cancer Hospital & Institute of Guangzhou Medical University, Gaozhou People’s Hospital, and The Second Affiliated Hospital of Zhengzhou University in 2 provinces (Henan and Guangdong) of China. All patients underwent TUR of bladder tumors, which was confirmed by cytology, before initiation of intravesical therapy. Inclusion criteria included Eastern Cooperative Oncology Group performance status < 2, age > 18 years, and intermediate and/or high risk NMIBC as defined by the 2001 European Association of Urology definitions. Pathological stage included any pT1 urothelial carcinoma (UC) and/or CIS and/or pTa lesions. The exclusion criteria were low risk NMIBC; histology other than UC; pathological stage T2 or higher; previous intravesical treatments; pelvic radiotherapy, systemic chemotherapy, or partial cystectomy; residual urine > 100 mL; bladder volume < 150 mL; persistent haematuria; active intractable or uncontrollable urinary tract infection; hematological disorders; kidney or liver function disorders (> 1.5 times upper normal limit); and pregnancy or lactation. A standard data form was created to retrieve relevant clinical, histological, and treatment-related data. In accordance with the precepts established by the Helsinki Declaration, all included patients were treated according to protocols approved by the respective institutional ethics committees.

### Apparatus and treatment regimens

**Figure 1:**
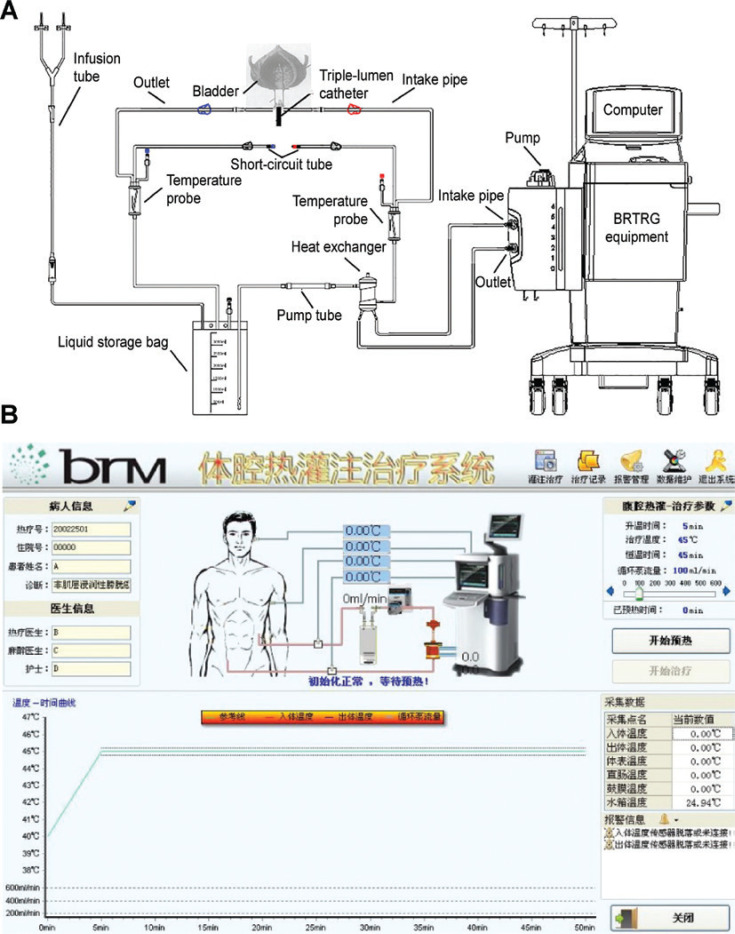
Schematic of the BRTRG urinary bladder hyperthermia treatment system (A) and a screenshot of the interface of the intelligent software (B).

The BRTRG urinary bladder hyperthermia treatment system is a cavity thermal perfusion system developed independently in China (Guangzhou Bright Medical Technology, Guangzhou, China). Our team contributed to the design and development of the device, which included 2 parts: a dedicated disposable perfusion tube and equipment with intelligent capabilities. These two parts comprised a continuously circulating closed circuit through a 3-way Foley catheter. The dedicated disposable perfusion tube included a dual ultrafiltration system. The cancer cells could be filtered effectively without flowing back to the bladder. The inlet pipe was connected to a chamber of the 20 Fr 3-way Foley catheter, and the outlet pipe was connected to another chamber. Each of the 2 pipelines was equipped with temperature probes (**Figure 1A**). The equipment used a computerized numerical heating control system, an automatic safety guarantee system, a non-interference temperature measuring system, and an active automatic cooling system. These technologies made use of water bath heating and high efficiency heat exchange to accurately control the temperature to within ± 0.1 °C of the target. The software used a fuzzy control algorithm to precisely regulate system operations and had a user-friendly interface, which included the patient’s clinical data, temperature, treatment time, and flow and power displayed in real time (**Figure 1B**).

The treatment choice was based on discussions by physician groups and was not randomized. All patients agreed and signed written informed consent forms before surgery and histological examination of the resected tissue. TUR was conducted with conventional procedures with a resection range of 2 cm of normal carrier tissue near the tumor, deep into the muscle layer. If there was no bleeding within 24–48 h after TUR, the patients were treated with IVEC or HIVEC. The bladder infusion chemotherapy drugs were MMC (20–60 mg) or pirarubicin (THP, 30–80 mg). In the IVEC group, the drug was dissolved in 50 mL of sterile water prior to injection into the bladder with a 20 Fr 3-way Foley catheter at room temperature, and the fluid was released after 60 min of retention. During the treatment, the patient changed position every 15 min. The induction treatment regimen was once a week for a total of 8 times, followed by once a month for a total of 10 times. Patients in the HIVEC+IVEC group underwent 3 consecutive CHT sessions followed by intravesical instillation without hyperthermia. The HIVEC session consisted of 45 min of continuous hyperthermia perfusion with 500 mL of mixed liquid (a chemotherapy drug and sterile water for injections) at 45 ± 0.1 °C. The amount of solution in the bladder was maintained at 300 mL, and the circulation flow rate was 100 mL/min. The perfusion fluid was drained from the bladder at the end of the treatment. These 3 sessions were conducted 48 h apart, and the subsequent IVEC treatment regimen was once a week for a total of 7 times, followed by once a month for a total of 10 times.

### Follow-up

Patients were followed-up every 3 months during the first 2 years, every 6 months until 5 years, and then annually. The follow-up visits included blood analysis, urinalysis, cytology, cystoscopy, and biopsies of suspicious areas. During every treatment or follow-up, any adverse events (AEs) were assessed and recorded according to the guidelines set by the National Institutes of Health, US Department of Health and Human Services (Adverse Events Record CTCAE V4.0).

### Statistical analysis

Statistical analyses were performed with SPSS statistical software for Windows, version 23.0 (SPSS, Chicago, IL, USA). Values for all continuous variables were expressed as the mean ± standard deviation (SD). Propensity score matching was used for matching cases. We compared parametric variables using *t*-tests and categorical variables using the χ^2^ test. Complications within the groups were evaluated by Fisher’s exact test. Safety results are presented in a forest plot portraying the odds ratio of each AE and the 95% confidence interval (CI) with a reference line indicating no effect [odds ratio (OR): 1]. Kaplan-Meier curves were constructed for each group. A significant difference was defined as *P* < 0.05.

## Results

### Patient characteristics

**Figure 2:**
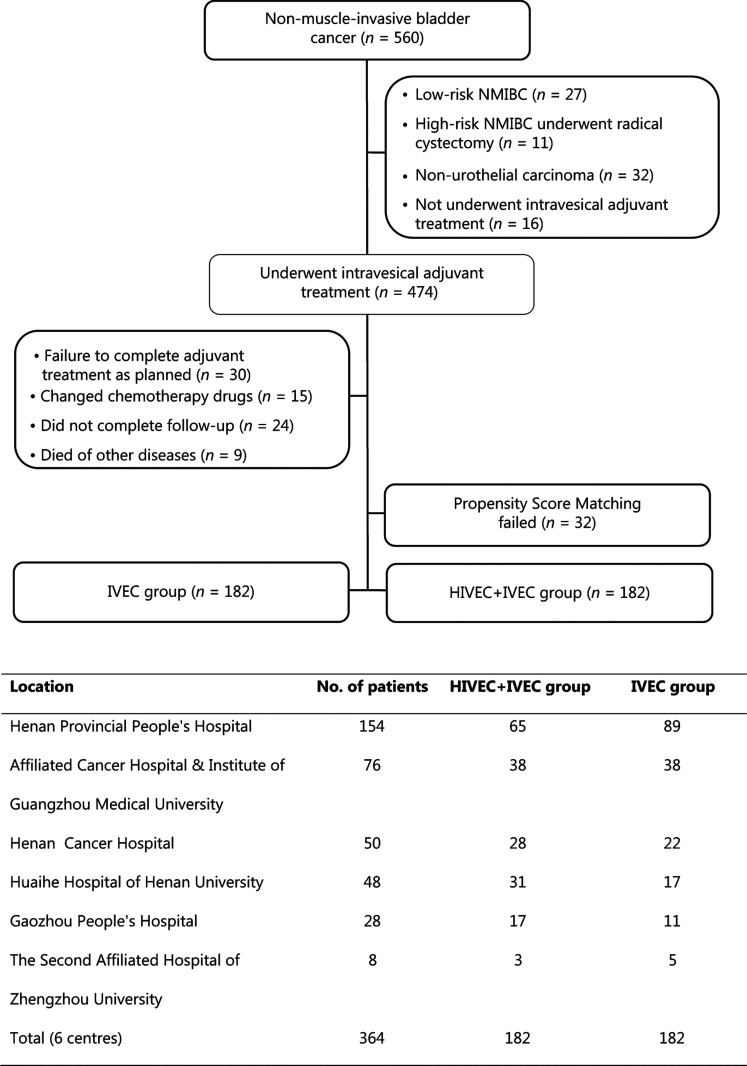
Flowchart of NMIBC patients and the number of cases at each center. IVEC, intravesical chemotherapy; HIVEC, hyperthermic intravesical chemotherapy; NMIBC, nonmuscle-invasive bladder cancer.

From April 2009 to December 2015, there were 560 patients diagnosed with primary or recurrent NMIBC at 1 of 6 tertiary centers. Of these 560 patients, 27 with low risk NMIBC and 11 patients with high risk NMIBC underwent RC prior to the availability of the pathological results. Thirty-two cases of non-UC were confirmed by postoperative pathology. Sixteen patients did not receive intravesical adjuvant treatment after TUR, and 30 patients did not complete adjuvant treatment as originally planned. Fifteen patients changed chemotherapy drugs during the treatment because of intolerance or financial reasons. Twenty-four patients did not complete follow-up, and 9 patients died due to non-cancer causes. After we excluded 32 cases with failed propensity score matching data, we successfully matched 364 NMIBC cases and divided them into the IVEC group (*n* = 182) and HIVEC+IVEC group (*n* = 182) (**Figure 2**). There was no significant difference in terms of clinicopathological data between the IVEC and HIVEC+IVEC groups (**Table 1**).

**Table 1:** Characteristics of patients (*n* = 364)

Characteristic	All patients	IVEC group	HIVEC+IVEC group	*P* value
Total	364	182	182	
Gender				0.794
Male	290	146	144	
Female	74	36	38	
Age				0.332
65 or less	225	117	108	
Greater than 65	139	65	74	
Other disease				0.261
Yes	116	53	63	
No	248	129	119	
Smoking history				0.382
Yes	130	61	69	
No	234	121	113	
Tumor size				0.393
Less than 3 cm	243	121	122	
3 cm or greater	62	35	27	
Missing	59	26	33	
Grade				0.475
G1	74	39	35	
G2	141	77	64	
G3	137	65	72	
Stage				0.406
T1	166	81	85	
Ta	186	99	87	
Risk group				0.284
High	144	67	77	
Intermediate	220	115	105	
Risk group (without CIS)				0.348
High	124	68	56	
Intermediate	208	103	105	
CIS				0.102
Pure CIS	12	2	10	
Papillary with CIS	20	9	11	
Chemotherapy drugs				0.166
Mitomycin	106	47	59	
Pirarubicin	258	135	123	

### Efficacy

**Figure 3:**
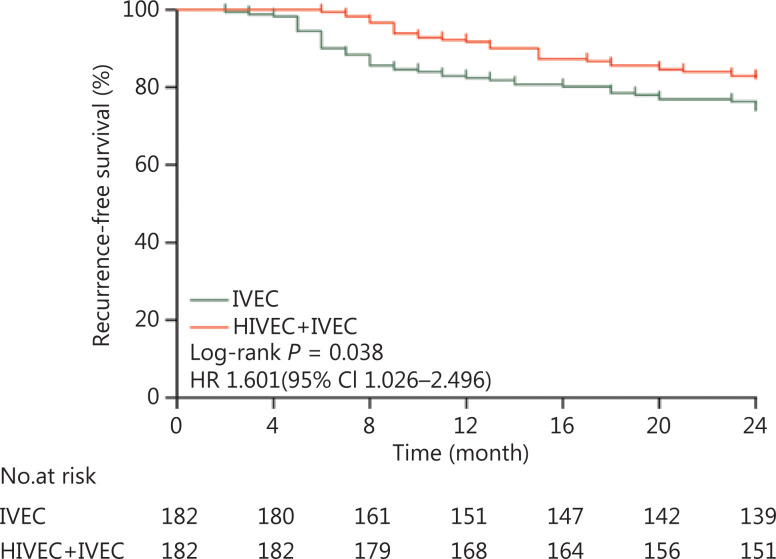
Kaplan-Meier curves for the recurrence-free survival of intermediate and high risk nonmuscle-invasive bladder cancer patients (*P* = 0.038). HR, hazard ratio; CI, confidence interval.

The median follow-up time was 63 months (range, 14–120 months) for all patients. The 2-year recurrence-free survival (RFS) was 82.42% (95% CI, 79.9%–84.9%) in the HIVEC+IVEC group and 74.18% (95% CI, 70.7%–77.8%) in the IVEC group (*P* = 0.038) (**Figure 3**).

**Figure 4:**
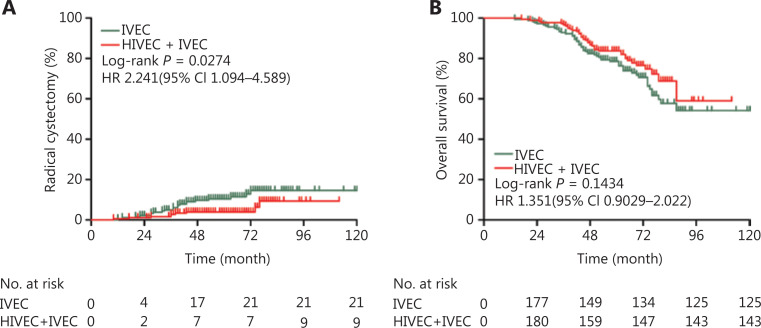
Incidence of bladder radical cystectomy (A) and overall survival (B) of the IVEC and HIVEC+IVEC groups estimated using the Kaplan-Meier method. The log-rank test was used to compare radical cystectomy and overall survival between the two groups. HR, hazard ratio; CI, confidence interval; IVEC, intravesical chemotherapy; HIVEC, hyperthermic intravesical chemotherapy.

The incidence of radical cystectomy (RC) in the IVEC alone group was higher than that in the HIVEC+IVEC group, and the difference was statistically significant (*P* = 0.0274, **Figure 4A**). Although there were more disease-related deaths in the IVEC group (*n* = 57) than in the HIVEC+IVEC group (*n* = 39), there was no significant difference in the 5-year overall survival (OS) between the two groups (*P* = 0.1434, **Figure 4B**).

### Safety

**Figure 5:**
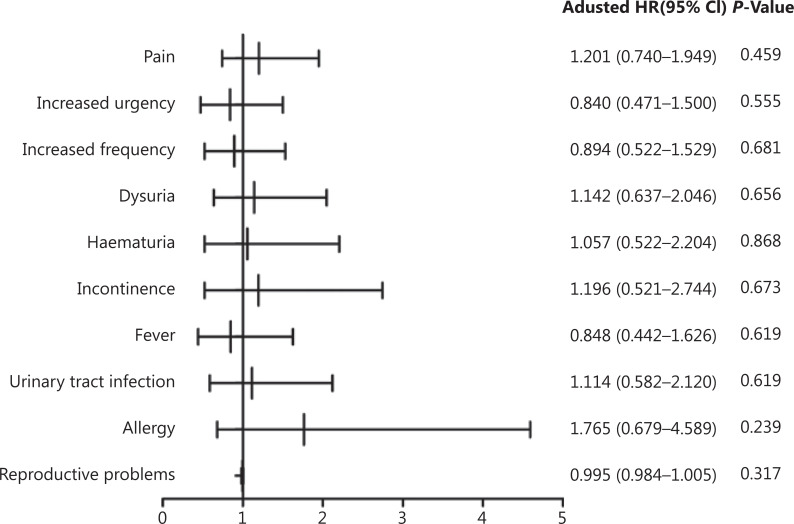
Forest plot showing hazard ratios and 95% confidence intervals for adverse events. CI, confidence interval; OR, odds ratio.

AEs were recorded for patients who underwent at least 1 treatment (*n* = 364) and included pain, increased frequency of urination, increased urgency, dysuria, hematuria, incontinence, fever, urinary tract infection, allergy, and reproductive problems (**Table 2**). One or more AEs occurred in 119 (32.7%) patients (54 in the IVEC group *vs.* 65 in the HIVEC+IVEC group). All AEs were grades 1–2, and no serious AEs (grades 3–4) were reported. In the HIVEC+IVEC group; the most prevalent AEs during treatment sessions were pain (*n* = 40, 22.0%) and increased urgency (*n* = 29, 15.9%), whereas the most prevalent AEs after treatment were increased frequency (*n* = 34, 18.7%), dysuria (*n* = 25, 13.7%), fever (*n* = 22, 12.1%), hematuria (*n* = 20, 11.0%), urinary tract infection (*n* = 20, 11.0%), incontinence (*n* = 11, 6.0%), allergy (*n* = 7, 3.8%), and reproductive problems (*n* = 2, 1.1%). In the IVEC group, the most prevalent AEs during treatment sessions were pain (*n* = 46, 25.3%) and increased urgency (*n* = 25, 13.7%), whereas the most prevalent AEs after treatment were increased frequency (*n* = 31, 17.0%), dysuria (*n* = 28, 15.4%), urinary tract infection (*n* = 22, 12.1%), hematuria (*n* = 21, 11.5%), fever (*n* = 19, 10.4%), incontinence (*n* = 13, 7.1%), allergy (*n* = 12, 6.6%), and reproductive problems (*n* = 3, 1.6%). No difference in overall AEs between each treatment modality was observed (**Figure 5**).

**Table 2:** Reported adverse events stratified by treatment

Adverse events	IVEC group (%) *n* = 182	HIVEC+IVEC group (%) *n* = 182
Pain	46 (25.3%)	40 (22.0%)
Increased urgency	25 (13.7%)	29 (15.9%)
Increased frequency	31 (17.0%)	34 (18.7%)
Dysuria	28 (15.4%)	25 (13.7%)
Hematuria	21 (11.5%)	20 (11.0%)
Incontinence	13 (7.1%)	11 (6.0%)
Fever	19 (10.4%)	22 (12.1%)
Urinary tract infection	22 (12.1%)	20 (11.0%)
Allergy	12 (6.6%)	7 (3.8%)
Reproductive problems	3 (1.6%)	2 (1.1%)

## Discussion

Approximately 2.7 million patients worldwide have been diagnosed, treated, and followed for bladder cancer at any given time point^20–22^. TUR is the primary treatment for NMIBC, but it cannot overcome the issues of high recurrence and progression. Therefore, other treatments must be given after TUR^23^. Early studies reported that IVEC can reduce and delay NMIBC recurrence^6,7^. Kato et al.^24^ reported that long-term intravesical instillation with epirubicin plus Ara-C combined with TUR improved the 5-year RFS by 19.9% compared with TUR alone in low grade superficial bladder cancers. In recent years, it has been reported that CHT is more advantageous than intravesical instillation alone. One of the most promising strategies is intravesical instillation of MMC by CHT, which compared with IVEC alone, improved RFS without increasing the incidence of local or systemic side effects^25^. Lammers et al. performed a meta-analysis in 2011 and found a 59.0% relative decrease in recurrences after CHT with MMC when compared with MMC alone^26^. However, clinicians and researchers who published papers on this therapy usually used microwave-generating devices, which exhibited efficient circulation of the fluidics but could not accurately control the circulation temperature. In a recent report in which 21 patients received MMC plus microwave-based hyperthermia, the treatment was poorly tolerated, and approximately 38% of the patients required therapy cessation. At a median of 50 months, 29% of the patients remained free of recurrence^27^. The BRTRG system can overcome these shortcomings and reduce microwave radiation exposure to the patient and medical staff. This device comprises a console, a peristaltic pump, and a heat exchanger. Using the heat exchanger, the perfusion fluid is heated and constantly cycled through the patient’s bladder.

CHT is different from previous chemotherapies without temperature management, because it efficiently combines hyperthermia and chemotherapy. CHT uses thermodynamic effects to increase the efficacy of anticancer drugs. The treatment results have been confirmed by many experimental and clinical reports. Colombo et al.^17^ conducted a prospective, multicenter, randomized study between 1994 and 2000 in which 83 NMIBC patients were randomized to receive HIVEC or MMC alone after complete TUR. CHT uses an intravesical microwave applicator to deliver hyperthermic fluids to the bladder. The MMC solution (40 mg in 50 mL) was heated to a medium temperature of 42.0 ± 0.2 °C for at least 40 min. Both groups of patients underwent an induction cycle consisting of 8 weekly sessions and a subsequent maintenance regimen of 4 monthly sessions. The results indicated that tumor recurrence in the MMC alone group occurred significantly earlier and more frequently than that in the CHT group. Witjes et al.^28^ reported that in patients with primary or BCG-failed CIS, the initial complete response (CR) percentage after intravesical hyperthermia and MMC was 92%, and the 2-year response percentage was approximately 50%. At the molecular level, CHT decreases proliferative activity and p53 activity^29^. Overall, these clinical and basic science studies showed that CHT was a promising treatment that could be more effective in reducing recurrence and improving survival for NMIBC.

Based on the database, we extracted the data of 560 NMIBC patients and matched 364 intermediate and high risk NMIBC cases. The 2-year RFS, incidence of RC, and 5-year OS were analyzed. Our study suggested that HIVEC+IVEC therapy had a higher 2-year RFS and a lower RC rate than IVEC therapy in intermediate and high risk NMIBC patients. A multicenter trial was conducted in 83 patients with NMIBC and showed that when compared with the MMC-only group, the MMC plus microwave hyperthermia group had lower disease recurrence (57.5% *vs*. 17.1%)^17^. Moskovitz et al.^30^ studied patients with intermediate or high risk recurrent superficial bladder cancer who were treated with intravesical MMC and local hyperthermia following surgery. Combined CHT ablated visible tumors in patients with an 80% CR during a mean follow-up of 104.5 days. Moskovitz et al.^31^ reported the results from their 10-year experience with CHT, showing that the disease-free percentage was 67.2%, and the tumor recurrence percentage was 28% within 2 years. A randomized controlled trial suggested that CHT therapy had a higher 2-year RFS than BCG therapy in patients with intermediate and high risk papillary NMIBC^32^.

Regarding the safety data of this report, AEs were the same between the two groups in comparison with previous reports^33–35^. In general, pain and increased urgency were the most prevalent AEs during treatment sessions, whereas increased frequency and dysuria were the most prevalent AEs after treatment. Both treatments were similarly well-tolerated.

This dataset comprised 364 matched patients with intermediate and high risk NMIBC to identify treatments. Long-term surveillance for NMIBC (with the advanced age of patients at onset) is extremely difficult. Due to the absence of follow-up and treatment data in some cases, there was a deviation in the comparison of OS between the two groups. Data from the database were retrospective and offered a limited scope of patient information for analysis. This study was also subject to selection bias, and although we adjusted for all known patient factors, residual biases may remain.

## Conclusions

This retrospective study suggested that HIVEC+IVEC therapy had a higher 2-year RFS and a lower RC rate than IVEC therapy in intermediate and high risk NMIBC patients. No significant difference in OS was observed between the two groups, and both treatments were similarly well-tolerated. Based on these results, urologists might consider HIVEC+IVEC as an adjuvant treatment for intermediate and high risk NMIBC patients after TUR.
